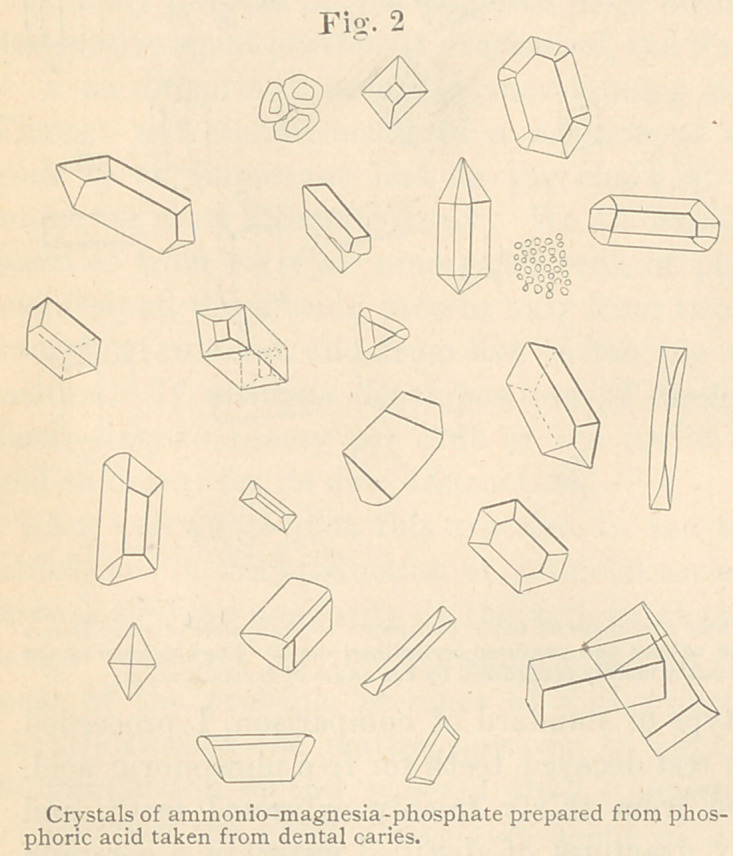# Dental Phosphorénèses, or Caries

**Published:** 1886-01

**Authors:** Edward S. Niles


### Independent Practitioner.

VOL. VII. JANUARY, 1886. No. 1.

### Original Communications.

### DENTAL PHOSPHORÉNÈSES, OR CARIES.

BY EDWARD S. NILES, D. M. D.

READ BEFORE THE HARVARD ODONTOLOGICAL SOCIETY AT BOSTON, FEB. 28, 1875; ALSO BEFORE THE MASSACHUSETTS AND CONNECTICUT VALLEY SOCIETIES AT WORCESTER, MASS., JUNE 24, 1885.

It is not always fair to hold a man to his own utterances, for it should be borne in mind that most men speak impulsively, and give expression or emphasis to that phase of a truth only which events have caused to be temporarily prominent before the mind. None of us stay keyed to a degree of watchfulness that can prevent all unguarded speech. Human nature must be vastly improved before the utterances of truth become universal, at least in the dental world, and the student will, doubtless, for generations to come, be obliged to select from a confusion of facts and improbabilities the knowledge that is to fit him for his sphere in life. Hence, the principal difficulty in dealing with the writings and discussions upon dental caries is the fact that the claims of those who have studied the subject involve part of the truth; those who oppose the theories advanced claim a whole error, and those who favor it the whole truth, and both become, in either case, impracticable theorists.

From year to year, as the result of earnest and faithful observation, some member of our profession presents a phase of the process of decay which should be allowed to remain before our minds, for without a system of records facts, do not accumulate. It is like an attempt to fill a bucket with water while there are holes in the bottom.

Close observation in the practice of past years has demonstrated many truths; many are self-evident, requiring no rigid experimental proof for acceptance; others, from circumstantial evidence, have been among the probabilities, and although not able to tell the why and wherefore, we have been more or less governed by them. There is, however, a universal feeling of applause among the members of our profession when a member makes a special study of the causes and progress of decay, and by experimental proof confirms our opinions. Inflammation, bacteria, putrefaction, fermentation, and various acids, are among the causes advocated and opposed. While we cannot select any one of these as fully explaining the phenomena of decay, we are forced to admit that each bears to us a part of the truth, and each, directly or indirectly, enters into and plays a part in this process. What earnest and honest student will not admit that inflammation is a factor in decay, and that the presence of micro-organisms has an influence upon this process? Who is prepared to wholly exclude many of the organic and mineral acids, or fermentation and putrefaction, with their products? The advocates of these theories occupy a prominent place in the profession, and when professional jealousies are no more, all will acknowledge their indebtedness.

Just at present, we are unusually interested in the experiments progressing at Berlin, conducted by Prof. Miller. His conclusions, I believe, are drawn from scientific reasoning, but though they bear the stamp that would lead us to accept all without hesitation, the sober after-thought makes us question just how much of the truth they bring to us. In justice to him, I will say that my criticisms are not based wholly upon my own experimental knowledge, or upon a repetition of all of his experiments, but partially upon those of well known authors, in and outside of our profession. Under the following six heads of conclusions printed in the May number of the INDEPENDENT PRACTITIONER, Prof. Miller sums up what he claims has been established and confirmed by him and others, in his method of study.

1st. "The observation of Leber and Rottenstein, that micro-organisms are constantly present in decaying dentine, has been confirmed. (Weil, Milles and Underwood, Miller.)"

Prof. Miller's experiments to confirm this are of great value.

2d. "The softening of dentine in caries has been shown to be chemically identical with that produced by certain weak organic acids. (Miller, Jeserich, Bennefeld.)"

I will affirm that the softening of dentine is *chemically identical* with that produced by any acid capable of removing the lime salts from dentine, which embraces a large number of organic and inorganic.

3d. "It has been established that various organisms found in the human mouth produce the decalcifying acid by first converting non-fermentable sugars into fermentable varieties; and secondly, by splitting fermentable sugars into lactic acid. (Miller, Hueppe.)"

It is not a new discovery that certain ferments, contained in saliva as well as other secretions of the body, have not only the power of converting cane sugar into grape sugar, but also of converting starch into the same sugar. It has also been generally understood that fermentable sugars may be converted into lactic acid, in the human mouth, by fermentation. The result of Magitot's experiments fairly established the presence of this process, and its effects upon tooth structure. Prof. Miller, however, deserves the credit of being more minute and exhaustive in his work. What he does affirm under this head, that is new, is that from sugar lactic acid is formed, and that this is the decalcifying agent in dental caries. It is possible lactic acid is generated from starches and sugars to an extent sufficient to respond to the zinc test, but the quantity produced no one has theoretically or experimentally shown. That lactic acid alone produces, or is the principal factor in decay, is very doubtful. It is further claimed that it is quite as possible to produce acetic acid from the varying fermentative elements and ferments of the mouth, as lactic acid.

4th. "The same organisms have been found capable of dissolving decalcified dentine, while they have no apparent effect, even after two or three years, on sound dentine. (Miller.)"

It is contrary to previous experimental teaching of the development of germs, that the same germinal ferment that converts unfermentable sugars into fermentable, and fermentable sugars into lactic acid (which must be of the acid variety), should also have the power to decompose albuminous tooth matrix, whose product would be alkaline, and require germs that propagate in that medium. Furthermore, this last action antagonizes the first by its alkalinity, and it is readily seen that this would neutralize the acid and arrest decay.

Unfortunately this is not the case, and many observations confirm the belief that the faster the matrix becomes exposed and softened, the more rapidly decay advances.

5th. "Caries of dentine, chemically and morphologically identical with natural caries, has been produced outside of the mouth. (Miller.)"

Dr. Miller has produced decalcification of dentine out of the mouth, in such a way that the matrix appears the same under the microscope as that decalcified in the mouth. Chemically, decay is the removal of the carbonate and phosphate of lime and the phosphate of magnesium from the tooth by acids, which is a very easy thing to accomplish out of the mouth.

Morphologically, decay is quite an impossible thing to accomplish out of the mouth. A tooth may be decalcified, or decay set up out of the mouth, but the vital resisting power of the tooth (inflammation, zone of resistance) cannot be counterfeited.

It is these influences, combined with the decomposed elements of the tooth, that create the different shades of matrix left, and what we call "white," "dark," "brown" decay.

6th. "It has been further shown that certain of the organisms of the human mouth are capable of developing under exclusion of air, thus making it possible for them to propagate within the substance of the dentine. (Miller, Hueppe.)"

If the certain germs are the same that produce lactic acid from sugar in the substance of the dentine, they are far from their base of supplies, and will hardly be able to develop lactic acid from sugar, as, from last accounts, there was no sugar in dentine at points too far in the tooth for the penetration of air, and Prof. Miller does not believe in the destruction of dentine by the physical force of these germs.

In submitting the above criticisms, after a careful study of Prof. Miller's work, I do not wish to be understood as bringing any serious refutation of his claims, unless he has cleared or covered the ground to his own satisfaction. I understand his conclusions under these six heads to be final, and that he has so placed them before the profession.

I regard his experiments, leading up to the above conclusions, as the most rigid and comprehensive that the dental profession have had the honor of receiving from among its members, and in the main valuable, in that they establish what we have long felt true, and we have to thank him for this acquisition to our theory and practice of dentistry.

I cannot feel, however, that we have sufficient evidence to believe that the persistent acidity along the border line of decay, or between the line of sound dentine and the decalcified matrix, is due to lactic acid. The frequent location and progress of the trouble alone leads us to look for the cause within the tooth.

The presence of an acid reaction at the point indicated is unquestioned, and it must also be conceded as a product of fermentation. Prof. Miller has seen evidences of sugar fermentation, and detected lactic acid in caries. Whether he believes that the sugar element produces acid sufficient for the whole decalcification in caries, he does not affirm; he gives us no idea how great a quantity is produced in the tooth or mouth, and what portion of this is neutralized by an alkaline saliva, or what amount may possibly find its way to tooth substance to be neutralized by the lime of the tooth acting as an antacid.

Let us suppose, for a moment, that we have a closed, secluded point on the approximate surface of two superior bicuspids, at a small defective place in the enamel; it progresses rapidly, and although one-third of the tooth crown is destroyed, the enamel remains apparently intact, the only evidence of decay visible being the discoloration. Not even the finest instrument is admitted until the enamel is broken, and then we find a decalcified and softened matrix, which is strongly acid throughout. I might cite many instances of decay where there is no apparent place for the lodgment of sugars.

If the acid theory is correct, as previously presented, we must believe that the acids are either developed about the external surfaces of the teeth, and after development enter the cavity as such, or they are developed in the tooth from foreign materials which have entered the cavity as food, saliva, sugars, etc. It would seem if the acids are developed outside, its presence would be marked by greater destruction of the enamel.

In either case, we are left to surmise the force and source of material that maintains this condition, favoring decay in the cavity from without, also that which bears away the products of decalcification.

As far as we know, there are four sources of alkaline products, so located as to neutralize the acids generated, and thus positively antagonize decay.

1st. The vital resisting power of the tooth.

2d. The alkalinity of saliva.

3d. The alkaline products from the decomposing tooth matrix.

4th. The antacid effect of the lime salts of the tooth.

In the outward elements, what is the place of lodgment in a well kept mouth for starches and sugars from which to develop acids sufficient for the demand? and in the advanced stages of decay, how are they carried to the point of decalcification? At this time I do not wish to attempt to point out all the probabilities and improbabilities in the theories previously advanced, regarding the progress of decay. There is, however, sufficient evidence to believe that external agents contribute to the acid supply in the *primary* stages of decay, but in the advanced and more rapid progress of the trouble a greater source of decalcifying power is apparent.

It is my purpose in this paper to point out a possible source of a destructive agent previously overlooked, and if my tests are confirmed it is an important factor in maintaining the acidity of caries. Though not alone considered a solution of the whole trouble, it must be an important link in the chain of chemical changes to which we must attribute decay. We all know that teeth are composed of from sixty to ninety per cent. of phosphate of lime and magnesium, there being present only from two to three per cent. of fluoride of calcium, and from five to ten per cent. of carbonate of calcium. If ultimate decomposition of these salts were to take place, sixty to ninety per cent. of the tooth would be a source of acid sufficient for its own destruction.

I am well aware that this reaction in the tooth has been questioned, but in the production of artificial caries by phosphoric acid there is striking similarity of the various shades and conditions of decay. The test for free phosphoric acid is rendered difficult, because of the probable presence of a phosphatic salt. I have sufficiently studied the matter, however, to feel confident that, before the publication of this paper, I shall completely demonstrate and confirm that the great destructive agent of inorganic tooth structure is developed from the tooth itself, by the liberation of phosphoric acid by fermentation or putrefaction.

BOSTON, NOV. 11, 1885.

Since writing the above paper I have perfected my tests, and furnished what seems to me conclusive proofs of the presence of free phosphoric acid in human dental caries. So abundant is its presence, and so simple the test, that any dentist with an elementary knowledge of chemistry may verify the experiments for himself.

The following are my experiments:

A few drops of free phosphoric acid were placed in a test tube, with about six times its bulk of distilled water. Ammonia was added slightly in excess. To this was added sulphate of magnesia, after which ammonium chloride (sal ammoniac). This was left to crystallize for twenty-four hours, then with a glass rod, on which had been previously slipped a piece of rubber tubing, crystals were detached, placed upon a slide, and brought under a microscope of three hundred diameters or more. Fig. 1 accurately represents some of the forms of crystallization of ammonio-magnesium-phosphate found in the field.

Fig. 1

These specimens of triple phosphate crystals were selected from a large variety and confused crystallized mass. The character of the crystals is mainly recognized by the angle of crystallization.

Taking this for my type or standard of comparison, I proceeded in the following way to test decayed teeth for free phosphoric acid. The decay was removed from thirty freshly extracted teeth, and placed, with about forty grammes of distilled water, in a common earthen mortar, and well ground and stirred. This was allowed to stand twenty-four hours, and again stirred and filtered. The filtrate

(quite clear), on being testing, was found acid. (It reddened blue litmus.) Preliminary tests were first applied to this liquid in the following way: To a portion was added strong nitric acid until strongly acid. Into this was introduced a small crystal of molybdate of ammonium. A very marked precipitate of yellow phospho-molybdate of ammonia was soon observed, indicating the presence of free phosphoric acid, or a phosphate. As it was possible that the above precipitate might be due to the presence of the salts of phosphate of lime and magnesium held in solution by other acids, and not due to free phosphoric acid alone, the following confirmatory tests were applied:

The remainder of the above filtered solution was evaporated in a porcelain evaporating dish to one-half its bulk. To a portion of this was added pure ammonia hydrate (no precipitate occurred, indicating the absence of a phosphate.) The test was then continued as in producing the preceding standard of comparison. Ammonia was added slightly in excess, then several drops of sulphate of magnesium, also ammonium chloride (sal ammoniac), and the whole well shaken. A precipitate of ammonio-magnesia-phosphate formed slowly.

Fig. 2

Crystals of ammonio-magnesia-phosphate prepared from phosphoric acid taken from dental caries.

This solution was allowed to stand in a test tube for twenty-four or thirty-six hours to crystallize. With a small glass rod, on which was slipped a piece of rubber tubing, crystals were removed from the sides of the tube to a slide, and placed under the microscope of about three hundred diameters for examination. The field was literally filled with crystals, and unmistakably the triple phosphates referred to, as will be seen in the accompanying Fig. 2.

It will at once be seen that these crystals could not be formed without free phosphoric acid. Here are four distinct tests, and any one of the three last named sufficient to fairly prove the presence of free phosphoric acid. Then, if phosphoric acid is present in decay, there can be no other source for it than from the phosphates of the tooth. So abundant are the proofs of free acid, and the almost entire absence of phosphates, that I am led to believe that this acid alone is the main factor in tooth destruction, and beyond question responsible for the acid reaction so persistent along the border line between decay and sound tooth structure, the presence of acids derived from food, etc., playing a very insignificant, or no part at all, in deep-seated dental caries.

Under the light of the above experiments, it may be stated as reasonably true that decay in teeth progresses, not wholly because of an insufficient amount of lime salts, but rather because of incomplete organization of the phosphates and imperfect tooth development, rendering possible the decomposition or splitting up of tooth substance into its proximate principles.

Without submitting further experiments or expression of opinion, at this time, as to the probable agents that may influence the decomposition of phosphate of lime and magnesium into their proximate principles of lime, magnesium and phosphoric acid, or the part which the fluorides play in the process of decay, I present this, hoping it may furnish a basis for valuable experiments for those who may be now engaged in the study of the subject.

## Figures and Tables

**Fig. 1 f1:**
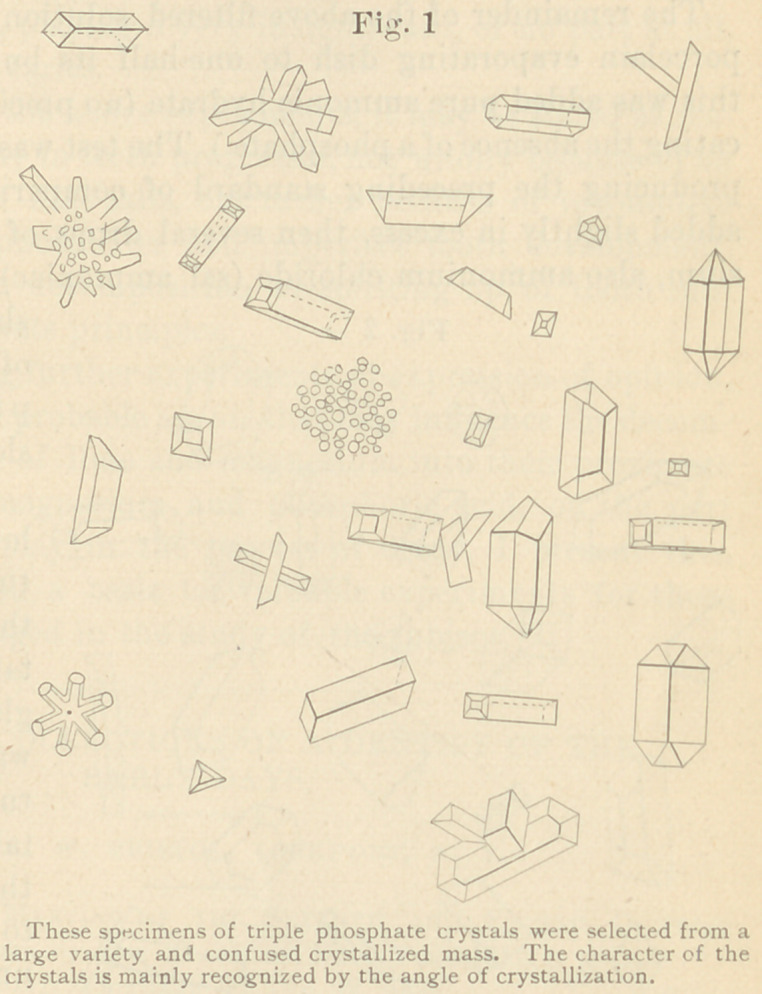


**Fig. 2 f2:**